# Comprehensive Analysis of SMC Gene Family Prognostic Value and Immune Infiltration in Patients With Pancreatic Adenocarcinoma

**DOI:** 10.3389/fmed.2022.832312

**Published:** 2022-03-15

**Authors:** Hui Nie, Yanhao Wu, Chunlin Ou, Xiaoyun He

**Affiliations:** ^1^Department of Pathology, Xiangya Hospital, Central South University, Changsha, China; ^2^Department of Respiratory Medicine, Xiangya Hospital, Central South University, Changsha, China; ^3^National Clinical Research Center for Geriatric Disorders, Xiangya Hospital, Central South University, Changsha, China; ^4^Departments of Ultrasound Imaging, Xiangya Hospital, Central South University, Changsha, China

**Keywords:** pancreatic adenocarcinoma, SMC family, immune infiltration, prognosis, biomarker, therapeutic target

## Abstract

Pancreatic adenocarcinoma (PAAD) is a malignant tumor with high morbidity and mortality worldwide. Members from the structural maintenance of chromosomes (SMC) gene family function as oncogenes in various tumor types, but their roles in PAAD have not been elucidated. In this study, we aimed to explore the role of the SMC family in tumor progression and cancer immune infiltration in PAAD using integrative bioinformatic analyses. The results showed that the SMC 1A, 2, 3, 4, and 6 were overexpressed in PAAD tissues; of these, SMC 1A, 4, 5, and 6 could be potential prognostic biomarkers for PAAD. The expression of SMC genes was found to be strongly associated with immune cell infiltration. According to the infiltrative status of various immune cells, the mRNA expression of *SMC* genes in PAAD was associated with the overall and recurrence-free survival of patients. In conclusion, the SMC gene family is associated with PAAD and may be involved in tumorigenesis and cancer-immune interactions; thus, members from this gene family may serve as promising prognostic and therapeutic biomarkers of PAAD.

## Introduction

Pancreatic adenocarcinoma (PAAD) is one of the most prevalent types of cancers worldwide, showing high mortality and transfer rates (1). The incidence of PAAD has recently increased, with 5-year survival rates of 2–9% (2). Because the clinical symptoms of PAAD are insidious and atypical, the diagnosis and treatment for this condition pose a critical challenge (3). Cancer cells usually disseminate by the time >80% of PAAD patients are initially diagnosed, making surgical removal ineffective (4). Early diagnosis and treatment are key to improving the prognosis of PAAD patients (5, 6). Therefore, new molecular markers and therapeutic targets are urgently needed to improve the clinical prognosis and outcomes of PAAD patients.

The structural maintenance of chromosomes (SMC) gene family comprises SMCs 1–6 (7, 8). While the SMC proteins are important for mitotic chromosome aggregation (9), the SMC1/3, 2/4, and 5/6 complexes play various biological roles in eukaryotes: the SMC1/3 complex mainly functions in chromosome cohesion, which can promote homologous recombination repair and aggregation, and affect transcription rates; the SMC2/4 complex is also closely associated with transcription as a condensation polymer; and the SMC5/6 complex helps in recombination, promotes replication, and affects mitotic chromosome allocation (10–15); the components of the SMC gene family have been linked to multiple tumors, suggesting their role in tumor occurrence and development. For example, SMC1 can promote epithelial-mesenchymal transition (EMT) in triple-negative breast cancer by inducing Brachyury expression (16). Feng et al. showed that the expression of SMC2 is closely related to PAAD (17). In addition, SMC2 can inhibit cytotoxicity, which promotes tumor sphere formation. Hence, a combination of anti-SMC2 antibodies, PTX, and 5-FU shows a strong potential for treating breast and bowel cancer (18). On the other hand, cohesion mutations associated with SMC3 may promote leukemia development by enhancing Wnt signaling (19), and SMC4 plays a key role in lung development and carcinogenesis. The overexpression of SMC4 can promote the proliferation of lung cancer cells and serves as an independent prognostic factor for lung cancer (20). The results of DNA microarray analysis have revealed the upregulation of SMC4 expression in PAAD, compared to the case in normal tissues, and thus, SMC2/4 may be a potential target for effective broad-spectrum anticancer agents (21). However, little is known about the effects of the SMC family of genes and proteins in the progression and pathogenesis of PAAD.

This study aimed to determine the function, and molecular mechanisms underlying the role, of SMCs in PAAD. We initially identified SMC expression in various types of cancer in the Gene Expression Profiling Interactive Analysis (GEPIA) database and then extended our analysis to PAAD using the Human Protein Atlas (HPA) and The Cancer Genome Atlas (TCGA) databases. Thereafter, we assessed the prognostic value of SMCs in PAAD using Kaplan–Meier curves and the mutation sites in the SMC genes identified using cBioportal. We also explored the potential SMC-linked pathways associated with PAAD and analyzed the SMC family-induced immune infiltration in PAAD by using the TIMER database.

## Materials and Methods

### Gene Expression Profiling Interactive Analysis

GEPIA is a site that provides fast and customizable functionality based on TCGA and GTEX data (22), from where we obtained the expression signatures of SMCs in various tumors. Further, we used the GEPIA database to analyze the expression of SMC family members in 171 normal pancreas samples and 179 PAAD samples.

### Human Protein Atlas Project

Although the main function of the HPA is primarily to explore the human proteome, we exploited it to assess the immune reactivity in tissues, the strength of individual cell populations, the fractions of immunoreactive cells, and the subcellular localization of the molecules (23).

### Kaplan–Meier Plotter

Kaplan-Meier plotter can assess the roles of 54,000 genes in 21 cancer types, based on the most comprehensive data available for breast, ovarian, lung, and stomach cancers (24). We evaluated the prognostic functionality of the SMC gene family in 177 patients with PAAD by linking TCGA data to their respective mRNAs using the plotter.

### cBioportal

cBioportal for Cancer Genomics enables the visualization, analysis, and download of large-scale cancer genomics datasets from 20 types of cancer and >5,000 tumor samples. It also provides rapid, intuitive, and high-quality access to the molecular profiles and clinical properties of large-scale cancer genomics projects (25, 26). We analyzed the SMC gene family using pancreatic cancer datasets of 168 patients from TCGA, the Pan-Cancer Atlas, and cBioPortal.

### Metascape

Metascape is a free gene annotation and analysis resource that helps investigators understand multiple gene lists and supports various enrichment analysis algorithms. It is convenient and enables data analysis in real time, and is thus, advantageous over other enrichment tools. We conducted the Kyoto Encyclopedia of Genes and Genomes (KEGG) pathway and Gene Ontology (GO) enrichment analysis for genes co-expressed with SMC members in PAAD.

### TIMER

TIMER aids the determination of relationships between gene expression and immune infiltration, and their effects on the disease prognosis (27). We studied the correlation between SMC gene expression in PAAD and immune infiltration. The correlation value R was calculated using the Spearman algorithm on the TIMER website and adjusted according to the tumor purity. Statistical significance was set at *p* < 0.05.

### Statistical Analysis

Gene expression data from the GEPIA database were analyzed using the Student's *t*-test. Correlations between the SMC gene family and clinicopathological features of PAAD were verified using χ^2^ test. Survival was analyzed by constructing Kaplan–Meier curves and log-rank tests using SPSS (SPSS version 25). Differences were considered statistically significant at *P* < 0.05.

## Results

### Multiple SMC Genes Were Upregulated During PAAD

**Figure 1:**
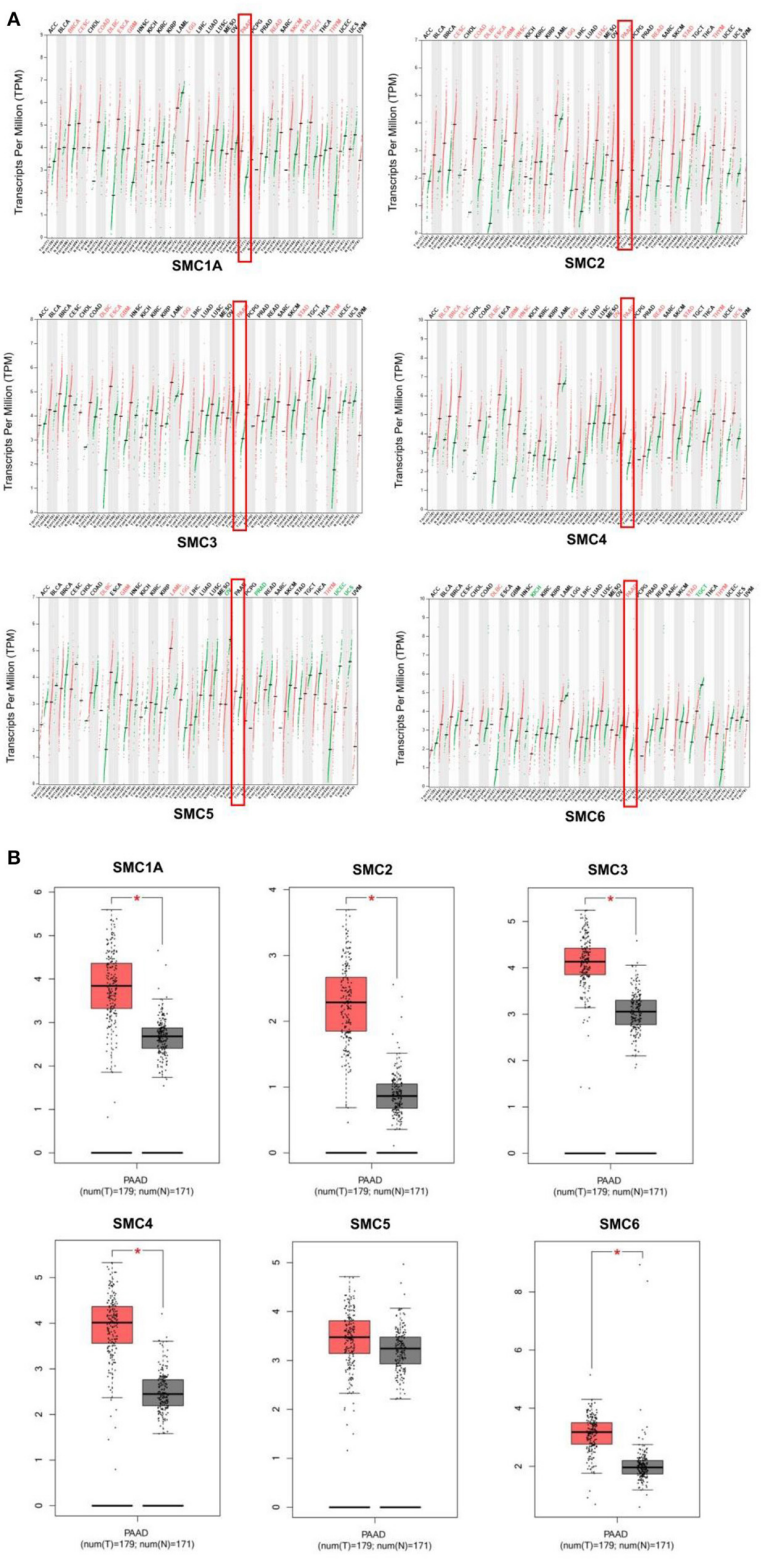
Differential expression of structural maintenance of chromosomes (SMC) family members. **(A)** The mRNA expression levels of SMC gene family members in different cancer types, as analyzed using the TIMER database. **(B)** The mRNA expression of SMC gene family members in pancreatic adenocarcinoma (PAAD), as obtained from the GEPIA database. ^*^P < 0.05.

**Figure 2:**
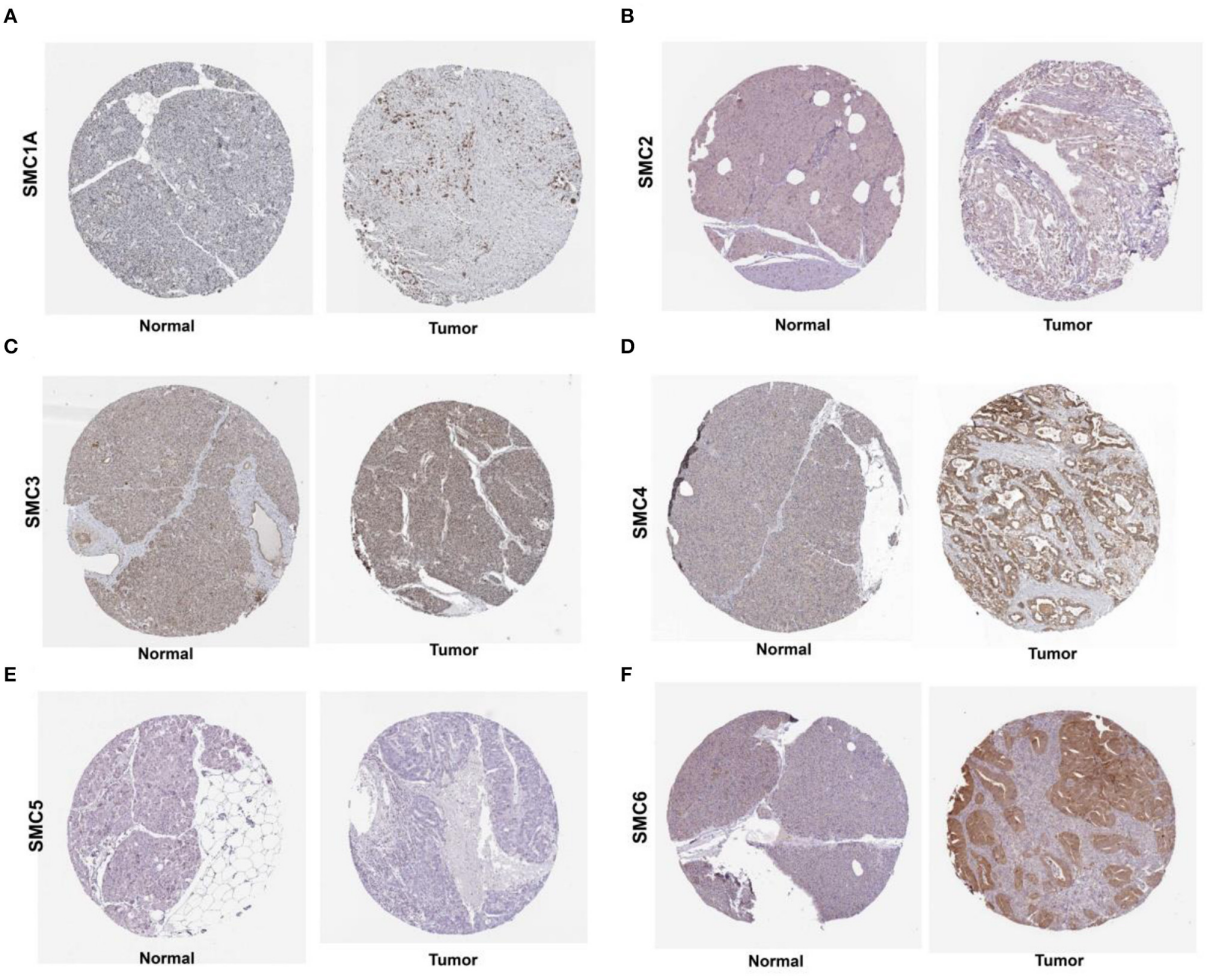
Levels of SMC proteins expressed in PAAD tissues, as obtained from the Human Protein Atlas (HPA). **(A–F)** Protein levels of SMC1A, 2, 3, 4, 5 and 6 in PAAD tissues, compared with those in noncancerous tissues (Original magnification, 40×).

We found that the mRNA expression of the SMC gene family was increased in most human cancers (Figure 1A). Likewise, the levels of SMC1A, 2, 3, 4, and 6 were elevated in PAAD compared to the normal tissues (revealed using GEPIA) (Figure 1B). We also detected their expression in PAAD using the immunohistochemical data from the HPA. The results showed an abundant expression of SMC1A, SMC4, and SMC6 in PAAD tissues; however, SMC2 was expressed weakly, compared to that in the normal tissues (Figure 2). On the other hand, the expression of SMC3 did not differ between PAAD and normal tissues and SMC5, which was weakly expressed in the normal tissues, was completely absent in the tumor tissues.

To draw correlations between the SMC gene family and the pathological characteristics of patients with PAAD, we downloaded the clinicopathological features of 156 patients from the TCGA database and examined the clinical relevance of SMC expression (Table 1). The results showed that the expression of SMC1A correlated with sex (*P* < 0.001), whereas that of SMC4 was significantly correlated with T stage, grade, and diameter (all *P* < 0.05, respectively). The expression of SMC2, 3, 5, and 6 was not associated with any clinicopathological parameters. These results indicate that SMC4 overexpression was significantly associated with advanced tumor stages, higher grade, and a large tumor size.

**Table 1:** Clinicopathologic parameters and the expression of SMC gene family members in PAAD.

		**SMC1A**			**SMC2**			**SMC3**			**SMC4**			**SMC5**			**SMC6**		
**Characteristics**	***N***	**Low**	**High**	***P***	**Low**	**High**	***P***	**Low**	**High**	***P***	**Low**	**High**	***P***	**Low**	**High**	***P***	**Low**	**High**	***P***
**Gender**				**0.000**			0.370			0.900			0.402			0.212			0.921
Male	86	66	20		54	32		50	36		50	36		43	43		46	40	
Female	70	26	44		39	31		40	30		36	34		42	28		38	32	
**Age**				0.749			0.625			0.623			0.158			0.339			0.399
≤ 60	51	31	20		29	22		28	23		24	27		25	26		25	26	
>60	105	61	44		64	41		62	43		62	43		60	45		59	46	
**T Stage**				0.688			0.366			0.499			**0.030**			0.902			0.296
T1 + T2	27	16	11		14	13		14	13		20	7		15	12		17	10	
T3 + T4	129	76	53		79	50		76	53		66	63		70	59		67	62	
**N Stage**				0.270			0.419			0.223			0.326			0.728			0.805
Nx + N0	44	30	14		24	20		22	22		27	17		23	21		23	21	
N1	112	62	50		69	43		68	44		59	53		62	50		61	51	
**M Stage**				0.327			0.191			0.027			0.244			0.184			0.727
Mx	81	51	30		52	29		52	29		48	33		48	33		46	35	
M0 + M1	76	42	34		41	35		38	38		38	38		37	39		38	38	
**Stage**				1.000			0.245			0.193			0.124			0.591			0.078
StageI	19	11	8		8	11		8	11		14	5		12	7		12	7	
StageII	130	77	53		81	49		79	51		67	63		70	60		71	59	
StageIII+IV	7	4	3		4	3		3	4		5	2		3	4		1	6	
**Grade**				0.836			0.390			0.459			**0.014**			0.994			0.781
Gx + G1	26	14	12		14	12		13	13		20	6		14	12		15	11	
G2	82	53	29		31	51		51	31		46	36		45	37		42	40	
G3 + G4	48	25	23		18	30		26	22		20	28		26	22		27	21	
**Diameter (cm)**				0.347			0.106			0.857			**0.045**			0.895			0.683
≤3	58	37	21		39	19		34	24		38	20		32	26		30	28	
>3	98	55	43		53	45		56	42		48	50		53	45		54	44	

*Bold font indicates significant difference*.

### Mutations in, and Possible Mechanisms Underlying the Action of, Genes From the SMC Family Underlying in PAAD

**Figure 3:**
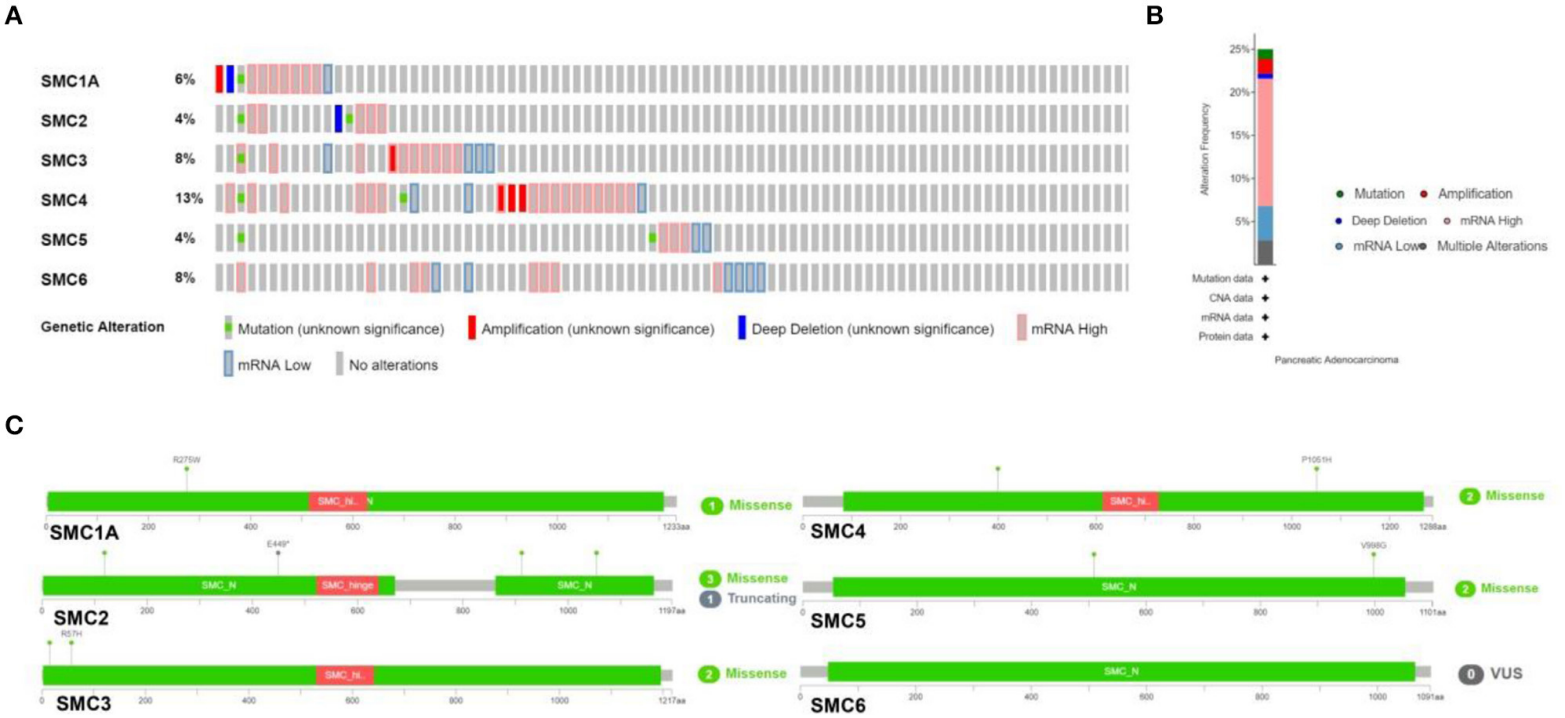
Mutations in SMC genes in PAAD, as determined using cBioPortal. **(A,B)** Summary of mutation frequency in SMC family genes in PAAD. **(C)** Specific types of mutations in individual SMCs.

We investigated genetic alterations in the SMC family in 168 samples from patients with PAAD using cBioPortal. The proportion of genetic variations in SMCs in patients with PAAD increased from 4 to 13% (Figure 3A). The proportions of genetic mutations were 4% in SMC2 and SMC5, 8% in SMC3 and SMC6, 6% in SMC1A, and 13% in SMC4. The most frequent abnormalities in the entire SMC family in PAAD patients comprised mRNA alterations, followed by amplification and other types of mutations (Figure 3B). Missense mutations were identified in SMC1A, 2, 3, 4, and 5 (Figure 3C), and two missense mutations were found in SMC4 and SMC5. Three missense mutations and one truncating mutation was found in SMC2. These results show highly heterogeneous genetic and expression changes in the SMC gene family in PAAD patients, suggesting that these genes play important roles in the PAAD microenvironment.

**Figure 4:**
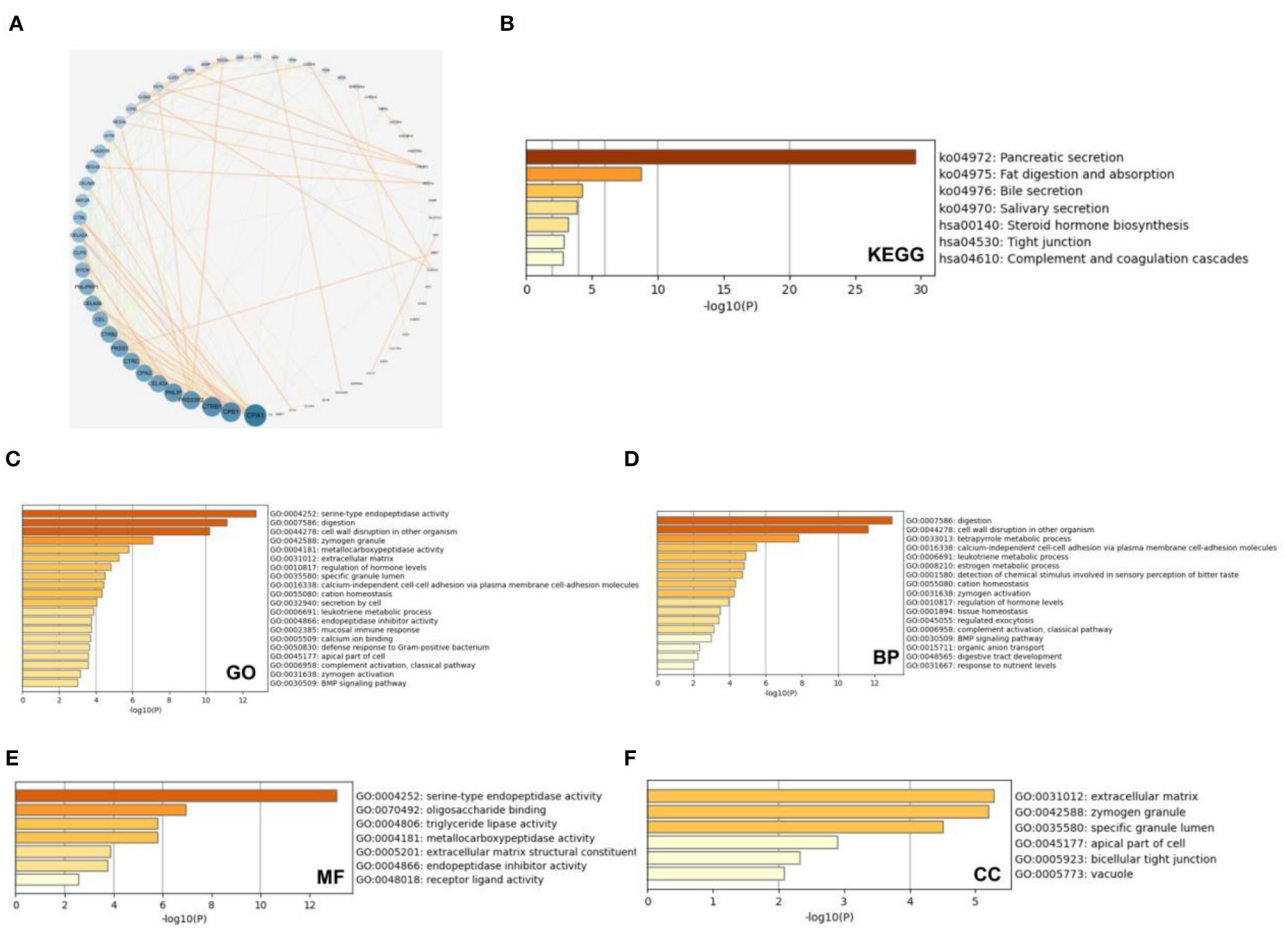
Enrichment analysis of SMC gene family and neighboring genes in PAAD tissues. **(A)** Gene—gene interaction network between SMC family genes and 132 co-expressed genes. **(B)** KEGG analysis of SMC family genes and co-expressed genes and **(C)** GO analysis of these genes based on **(D)** biological processes (BP), **(E)** molecular function (MF), and **(F)** cellular component (CC).

We analyzed the co-expressed genes and enrichment pathways of the SMC family in patients with PAAD. We initially downloaded 132 relevant genes with a threshold of *P* < 0.05, |log_2_FC| > 1.5, which were co-expressed with the SMC family in patients with PAAD (Figure 4A). Among them, CPA1 was the most closely correlated with the SMC gene family. We comprehensively investigated the biological functions of these genes by analyzing their GO terms and their KEGG pathways using Metascape database. The GO results showed that the co-expressed genes were primarily correlated with serine-type endopeptidase activity, digestion, cell wall disruption in other organisms, zymogen granules, and metallocarboxypeptidase activity (Figure 4B). Further, the KEGG results showed that the co-expressed genes primarily correlated with pancreatic secretion, fat digestion and absorption, bile secretion, steroid hormone biosynthesis, tight junction, and complement and coagulation cascades (Figure 4C). The top enriched GO pathways for biological process comprised digestion, cell wall disruption in other organism, tetrapyrrole metabolic processes, calcium-independent cell-cell adhesion via plasma membrane cell-adhesion molecules, leukotriene metabolic process, and estrogen metabolic process; the enriched terms for molecular function comprised serine-type endopeptidase activity, oligosaccharide binding, triglyceride lipase activity, metallocarboxypeptidase activity, extracellular matrix structural constituents, and endopeptidase inhibitor activity; and the enriched terms for cellular components comprised extracellular matrix, zymogen granule, specific granule lumen, apical part of cell, bicellular tight junction, and vacuole (Figures 4D–F). These results suggested that the SMC gene family is involved in the regulation of drug metabolism in PAAD, providing new possibilities for future drug therapies for PAAD.

### Correlations Between SMC Expression and Immune Infiltration in PAAD

**Figure 5:**
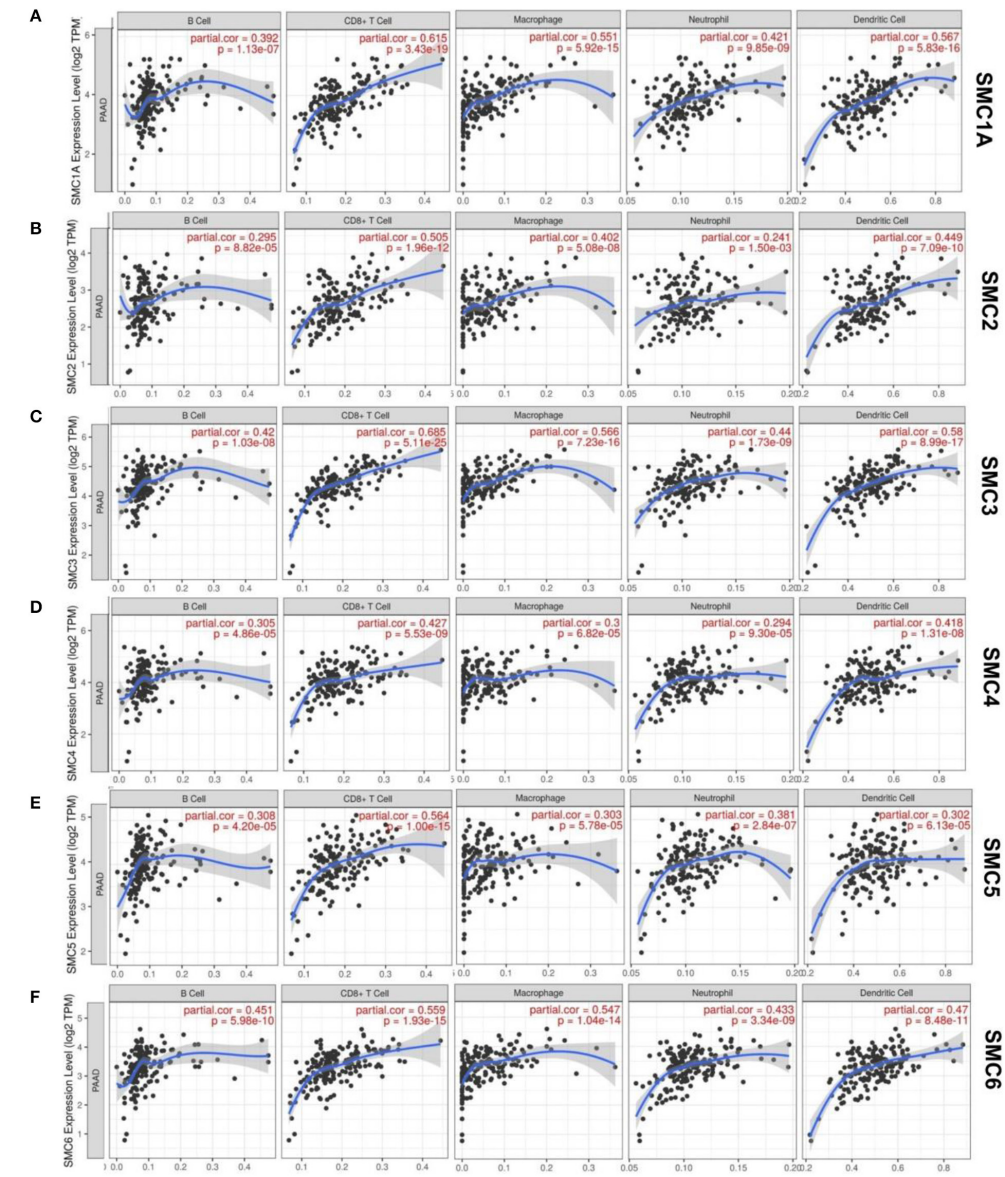
Relationships between SMC protein expression in PAAD patients and immune cell infiltration. **(A–F)** Correlation between the expression of SMC1A, 2, 3, 4, 5, 6 and the immune infiltration by macrophages, neutrophils B, CD8+ T, and dendritic cells.

Some possible pathways involving SMCs in patients with PAAD have been determined. Of these, few were involved in the immune infiltration during PAAD. We then investigated whether SMC gene transcription plays vital roles in the etiology of PAAD through immune cell infiltration (Figure 5). The TIMER results showed that the expression of SMC2 and SMC4 correlated with the infiltration of six types of immune cells (B cells, CD8+, and CD4+ T cells, dendritic cells, macrophages, and neutrophils) in PAAD, of which correlations were negative for B cell infiltration, and positive for other types of cells (*P* < 0.05). Moreover, B cell, CD8+ T cell, macrophage, neutrophil, and activated NK cell infiltration were positively correlated with the gene expression of SMC1A, 3, 5, and 6 (*P* < 0.05). Therefore, the results from the TIMER database showed that some SMCs are essential and unique in the PAAD tumor microenvironment.

We further explored relationships between the SMC genes and the diverse immune cell infiltrates in PAAD (Table 2). We found a close correlation between SMC1A and CD8+ T cells, M2 macrophages, Tregs, and monocytes, and between SMC2 and monocytes, as well as a moderate or weak correlation between SMC2 and CD8+ T cells, M2 macrophages, Th1, Tfh, Th17, and Tregs. A relatively close correlation was evident between SMC3 and CD8+ T cells, T cells (general), M2 macrophages, Th1, Tregs, and monocytes and a moderate relationship was identified between SMC4 and M1 and M2 macrophages, Th2, and Treg markers. Correlations were moderately close between SMC5 and CD8+ T cells, M1 macrophages, neutrophils, Th2 cells, Tregs, and monocytes, and very close between SMC6 and B cells, M2 macrophages, Th2, Tregs, and monocyte markers in patients with PAAD. A clear relationship between some components of the SMC gene family and monocyte and M2 macrophage markers notably suggested that the SMC gene family is involved in regulating macrophage polarization in patients with PAAD. In addition, all results indicated that SMC family members plausibly play critical roles in the immune infiltration in PAAD.

**Table 2:** The association between the expression of SMC family members and the markers of immune cells.

		**SMC1A**		**SMC2**		**SMC3**		**SMC4**		**SMC5**		**SMC6**	
		**Cor**	***P***	**Cor**	***P***	**Cor**	***P***	**Cor**	***P***	**Cor**	***P***	**Cor**	***P***
CD8 + Tcell	CD8A	0.338	***	0.204	**	0.371	***	0.091	0.238	0.268	***	0.335	***
	CD8B	0.333	***	0.199	**	0.345	***	0.099	0.197	0.189	*	0.262	**
	GZMA	0.269	***	0.184	*	0.275	***	0.067	0.386	0.166	*	0.269	***
B cell	CD19	0.165	*	0.076	0.326	0.166	*	0.020	0.794	0.073	0.344	0.273	***
	CD79A	0.183	*	0.104	0.174	0.183	*	0.032	0.673	0.080	0.296	0.276	***
	MS4A1	0.158	*	0.052	0.497	0.185	*	−0.034	0.663	0.100	0.195	0.264	***
T cell	CD3D	0.218	**	0.082	0.285	0.270	***	0.007	0.930	0.124	0.107	0.225	**
	CD3E	0.289	***	0.144	0.061	0.323	***	0.040	0.607	0.200	**	0.297	***
	CD2	0.300	***	0.145	0.059	0.336	***	0.045	0.561	0.205	**	0.297	***
TAM	CCL2	0.114	0.137	0.015	0.846	0.131	0.088	−0.094	0.220	0.087	0.257	0.058	0.448
	CD68	0.347	***	0.225	**	0.402	***	0.285	***	0.257	**	0.381	***
	IL10	0.284	***	0.150	0.050	0.295	***	0.143	0.062	0.123	0.109	0.362	***
M1	IRF5	0.228	**	0.078	0.309	0.237	**	0.190	*	0.179	*	0.421	***
	PTGS2	0.229	**	0.270	***	0.239	**	0.445	***	0.366	***	0.429	***
	NOS2	0.322	***	0.250	**	0.226	**	0.237	**	0.198	**	0.261	**
M2	MS4A4A	0.389	***	0.267	***	0.414	***	0.150	*	0.133	0.082	0.369	***
	CD163	0.461	***	0.352	***	0.502	***	0.266	***	0.223	**	0.431	***
	VSIG4	0.336	***	0.247	**	0.368	***	0.176	*	0.135	0.078	0.320	***
Neutrophils	ITGAM	0.310	***	0.151	*	0.293	***	0.184	*	0.179	*	0.316	***
	CCR7	0.257	**	0.120	0.117	0.263	**	−0.026	0.737	0.183	*	0.255	**
	SIGLEC5	0.419	***	0.241	**	0.394	***	0.166	*	0.188	*	0.378	***
DC	HLA-DQB1	0.266	***	0.164	*	0.290	***	0.141	0.066	0.087	0.259	0.174	*
	HLA-DPB1	0.346	***	0.152	*	0.331	***	0.059	0.445	0.134	0.081	0.292	***
	HLA-DRA	0.471	***	0.277	***	0.452	***	0.200	**	0.200	**	0.391	***
	HLA-DPA1	0.486	***	0.307	***	0.477	***	0.178	*	0.179	*	0.402	***
	ITGAX	0.137	0.074	0.013	0.865	0.131	0.087	0.120	0.119	0.151	*	0.243	**
	CD1C	0.242	**	0.054	0.484	0.274	***	−0.045	0.557	0.212	**	0.270	***
	NRP1	0.590	***	0.411	***	0.679	***	0.352	***	0.445	***	0.462	***
NK cell	KIR2DL1	0.130	0.091	0.058	0.452	0.140	0.068	0.066	0.389	0.010	0.896	0.117	0.127
	KIR2DL3	0.250	**	0.120	0.119	0.175	*	0.114	0.139	0.107	0.165	0.160	*
	KIR2DL4	0.275	***	0.277	***	0.232	**	0.277	***	0.220	**	0.210	**
	KIR3DL1	0.239	**	0.122	0.111	0.152	*	−0.013	0.870	0.082	0.286	0.037	0.630
	KIR3DL2	0.206	**	0.144	0.060	0.185	*	0.157	*	0.069	0.371	0.223	**
	KIR3DL3	0.173	*	0.130	0.089	0.095	0.216	0.145	0.058	0.056	0.469	0.162	*
	KIR2DS4	0.123	0.109	0.048	0.534	0.068	0.379	0.056	0.464	0.113	0.143	0.071	0.356
Th1	TBX21	0.236	**	0.152	*	0.288	***	−0.001	0.993	0.175	*	0.231	**
	STAT1	0.488	***	0.432	***	0.551	***	0.512	***	0.357	***	0.465	***
	STAT4	0.306	***	0.209	**	0.318	***	−0.033	0.664	0.186	*	0.253	**
	IFNG	0.244	**	0.203	**	0.294	***	0.209	**	0.123	0.109	0.141	0.066
Th2	STAT6	0.252	**	0.129	0.094	0.361	***	0.316	***	0.465	***	0.400	***
	GATA3	0.076	0.324	0.160	*	0.175	*	0.255	**	0.231	**	0.322	***
	STAT5A	0.344	***	0.199	**	0.351	***	0.177	*	0.236	**	0.445	***
Tfh	BCL6	0.375	***	0.238	**	0.434	***	0.447	***	0.557	***	0.416	***
	IL21	0.249	**	0.232	**	0.203	**	0.048	0.534	0.048	0.534	0.168	*
Th17	STAT3	0.632	***	0.458	***	0.623	***	0.367	***	0.580	***	0.584	***
	IL17A	0.204	*	0.185	*	0.183	*	0.057	0.456	0.167	*	0.063	0.410
Treg	FOXP3	0.348	***	0.162	*	0.355	***	0.157	*	0.159	*	0.373	***
	STAT5B	0.540	***	0.385	***	0.535	***	0.204	**	0.395	***	0.581	***
	CCR8	0.476	***	0.279	***	0.495	***	0.257	**	0.287	***	0.476	***
T exhaustion-cell	PDCD1	0.177	*	0.078	0.310	0.208	**	0.047	0.546	0.155	*	0.184	*
	CTLA4	0.234	**	0.106	0.169	0.274	***	0.090	0.242	0.138	0.072	0.265	***
	HAVCR2	0.350	***	0.222	**	0.384	***	0.182	*	0.156	*	0.351	***
	LAG3	0.267	***	0.242	**	0.194	*	0.146	0.057	0.138	0.073	0.117	0.128
Monocyte	CD86	0.397	***	0.271	***	0.426	***	0.206	**	0.162	*	0.354	***
	C3AR1	0.427	***	0.284	***	0.448	***	0.166	*	0.178	*	0.380	***
	CSF1R	0.441	***	0.279	***	0.457	***	0.143	0.062	0.177	*	0.340	***

*Correlation R value was calculated by Spearman's algorithm and adjusted by tumor purity*.

* *P < 0.05*.

** *P < 0.01*.

*** *P < 0.001*.

### Prognostic Value of SMCs in PAAD Patients

**Figure 6:**
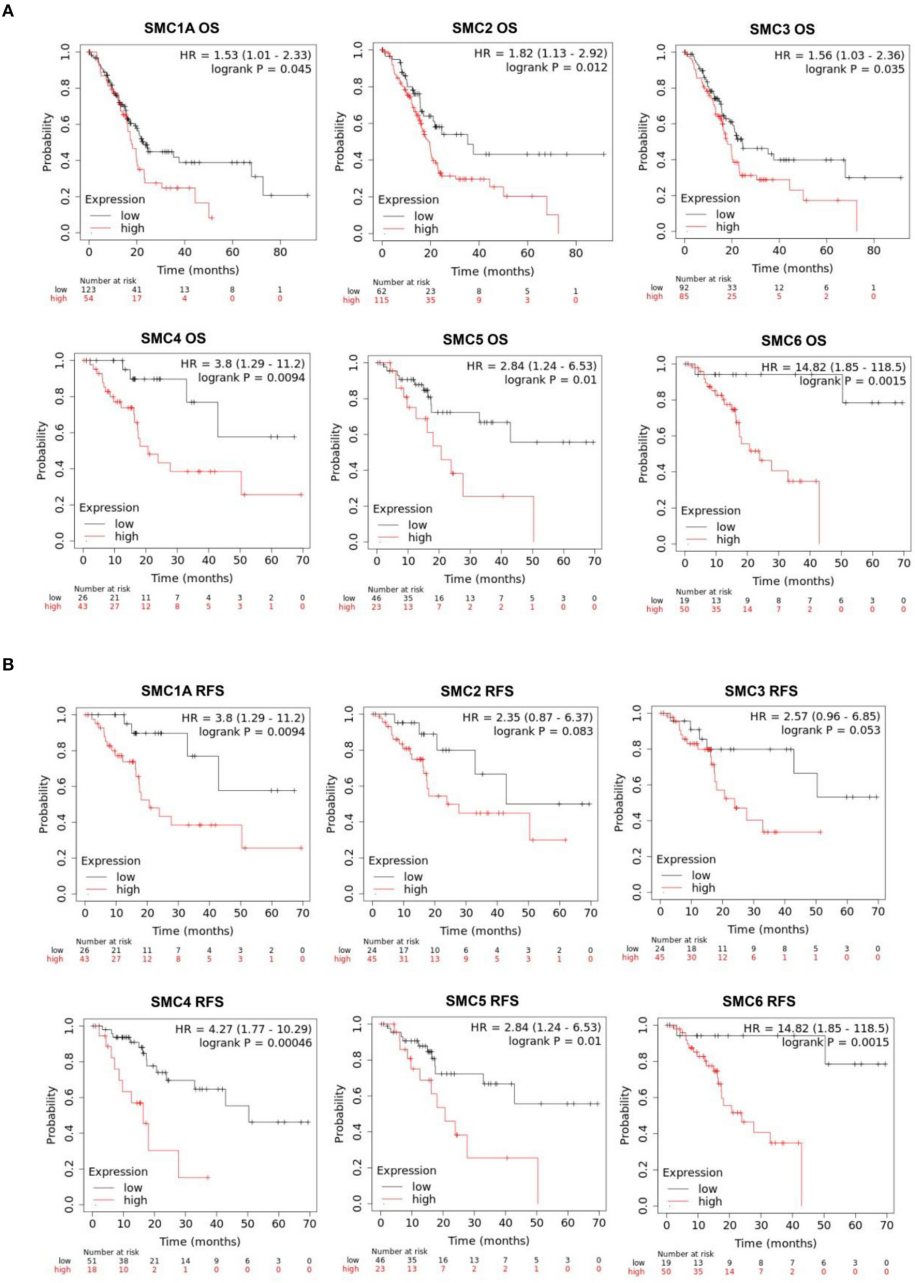
Prognostic value of SMC family gene expression levels with regard to the survival of PAAD patients. Relationships between SMC family gene expression levels and **(A)** overall survival (OS) and **(B)** recurrence-free survival (RFS) of PAAD patients, as estimated using Kaplan–Meier plotter.

The effects of SMC family expression on survival indices were evaluated using the Kaplan–Meier plotter. Increased SMC1A, 2, 3, 4, 5, and 6 expression levels were closely associated with poor overall survival (OS; *P* < 0.05; Figure 6A). An assessment of the relationship between individual SMCs and relapse-free survival (RFS) in patients with PAAD (Figure 6B) showed that SMC1A, 4, 5, and 6 were significantly correlated with the poor RFS. We concluded that SMC1A, 4, 5, and 6 could serve as potential biomarkers for the prognosis of patients with PAAD.

**Figure 7:**
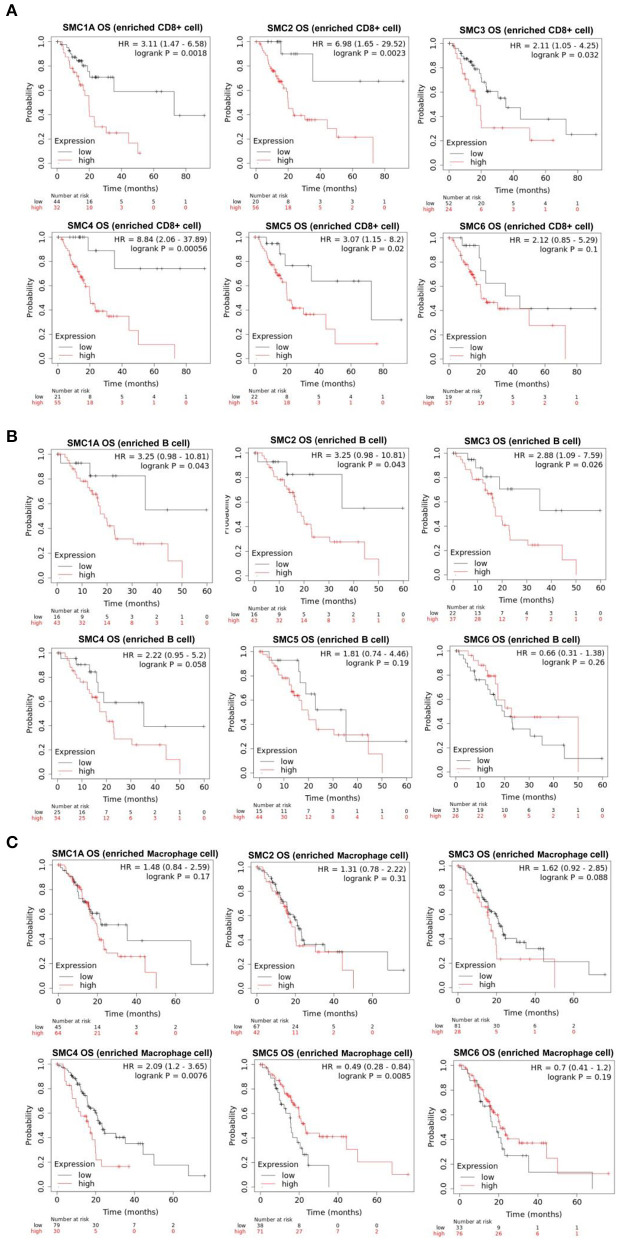
mRNA expression levels of SMC family genes in PAAD are associated with overall survival (OS). Relationships between the expression levels of SMC family genes and the OS of PAAD patients enriched with **(A)** CD8+ T cells, **(B)** B cells, and **(C)** macrophages.

**Figure 8:**
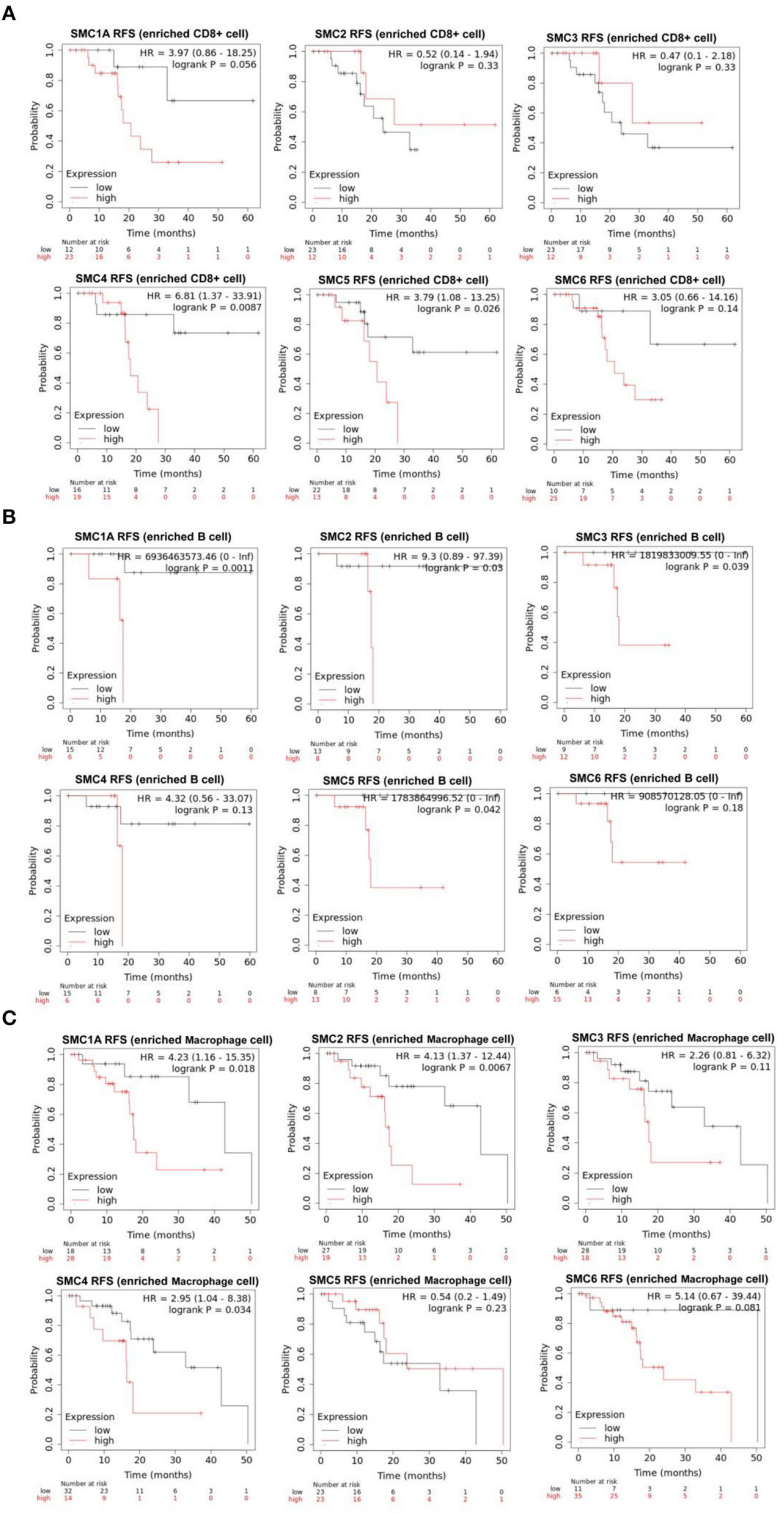
mRNA expression levels of SMC family genes in PAAD are associated with recurrence-free survival (RFS). Relationships between the expression levels of SMC family genes and the RFS of PAAD patients enriched with **(A)** CD8+ T cells, **(B)** B cells, and **(C)** macrophages.

The aforementioned results suggested that the SMC family members interact with immune cells and thus, play an important role in PAAD. Therefore, we further investigated the prognostic value of SMCs associated with immune cell enrichment in patients with PAAD. The results showed that upregulation of SMC1A, 2, 3, 4, and 5 was associated with the poor OS and decreased survival when PAAD patients were enriched with CD8+ T-cells (Figure 7A) and B-cells (Figure 7B). SMC4 expression was significantly and negatively correlated with OS only when PAAD patients were enriched in macrophages (Figure 7C). The upregulated expression of SMC1A, 4, 5, and 6 was strongly associated with an unfavorable RFS in PAAD patients with CD8+ T-cell enrichment (Figure 8A), and that of SMC1A, 2, 3, 5, and 6 was associated with poor RFS in PAAD patients with B-cell enrichment (Figure 8B). Upregulation of SMC1A, 2, 4, and 6 expression was associated with the unfavorable RFS in PAAD patients with macrophage enrichment (Figure 8C). These results suggested that immune cell infiltration is enhanced in patients with PAAD expressing an abundance of the SMC gene family, which help in improving their prognosis.

## Discussion

We systematically analyzed the expression and prognostic value of various SMC family proteins in patients with PAAD using bioinformatics tools. Upregulation of SMC1A is involved in the pathogenesis of various tumors, such as gliomas, and colon, triple-negative breast, and liver cancers (28–30). Moreover, SMC1A upregulation may be associated with the decreased OS of patients with colon and liver cancers (29, 30). However, low SMC1A expression in acute myeloid leukemia is associated with the poor prognosis (31). Enhanced SMC2 expression is associated with poorer OS in PAAD patients, suggesting that SMC2 is a crucial oncogene in PAAD (17). SMC2, when overexpressed, functions as an oncogene in bladder cancer (32). Additionally, frameshift mutations in this gene are associated with the pathogenesis of many cancers, including gastric and bowel cancers (17). Furthermore, SMC3 expression is upregulated in lung cancer, acute myeloid leukemia, and prostate cancer (32–34). The overexpression of SMC4 promotes glioma cell invasion by activating TGF-β/Smad signaling and is associated with poorer OS in patients with glioma (35). Similarly, SMC4 functions as a carcinogen and affects the OS of patients with breast cancer (36). The SMC5/6 complex plays a key role in alternative lengthening of telomeres (ALT) by maintaining telomere length via the SUMOylation of telomere-binding proteins (37). The relationship between HBx mutations and SMC5/6 in liver cancer cells, with regard to hepatitis B virus (HBV) infection, has received an increasing amount of attention (38, 39). We found significantly elevated SMC1A, 2, 3, 4, and 6 expression levels in PAAD tissues, compared with those in normal tissues. Increased mRNA expression levels of SMC1A, 2, 3, 4, 5, and 6 were associated with a shorter OS in patients with PAAD. Upregulated expression of SMC1A, 4, 5, and 6 mRNA was associated with decreased RFS among patients with PAAD. These results indicated that SMC1A, 4, 5, and 6 could be potential prognostic biomarkers for PAAD. Moreover, KEGG analysis of these members and their co-expression genes revealed the top 10 enriched pathways, among which drug metabolism pathways were significantly enriched, indicating that the members of the SMC gene family could serve as drug targets for PAAD. The GO studies showed that the role of the SMC gene family in PAAD is closely associated with human immune-related pathways.

PAAD is highly malignant and metastatic. The current primary treatment for this condition is surgery, in addition to radiation and chemotherapy, but the OS remains low. The advent of immunotherapy has brought new hope to patients with pancreatic cancer. Immune checkpoint molecules (PD-L1, CTLA-4, and PD1) inhibit the activity of T cells in the tumor microenvironment, thereby suppressing anti-tumor immunity-associated processes (40, 41). Enhanced T cell infiltration, together with a checkpoint blockade, may enhance the therapeutic effects in patients with PAAD (42). As active components of the tumor microenvironment (TME), B cells produce adaptive and anti-tumor immune responses, stimulate immune factors, and play an important role in the anti-tumor processes (43). Moreover, therapeutic vaccines may induce a powerful anti-tumor immune response, and the depletion of regulatory T cells (Tregs) can reduce TME suppression and help achieve considerable therapeutic effects (44). Therefore, immune-related therapies, combined with surgery, radiotherapy, chemotherapy, and targeted therapy, are promising treatments for PAAD (45, 46).

The present study revealed relationships between SMC family genes and immune infiltration in PAAD. We found that the expression of SMC family genes was significantly related to immune cell infiltration, indicating that SMCs could reflect the immune status of PAAD patients. In addition, enriched infiltration by anti-tumor immune cells, such as macrophages, CD8+T, and B cells, will improve the prognosis of patients with PAAD. Our findings provide sufficiently detailed information about immunization and will assist the design of new immunotherapies. Nevertheless, further improvements are needed, as the data analyzed herein were sourced online, and *in vitro* studies, as well as clinical investigations, are needed to confirm the potential mechanisms underlying the action of, molecular interactions among, and clinical applications of, various SMC genes in PAAD.

## Data Availability Statement

The original contributions presented in the study are included in the article/supplementary material, further inquiries can be directed to the corresponding authors.

## Author Contributions

HN and YW for acquisition of data, analysis and interpretation of data, statistical analysis, and drafting of the manuscript. CO and XH conceived and designed the experiments. All authors participated in writing the paper and read and approved the final manuscript.

## Funding

This study was supported by the National Natural Science Foundation of China (No. 81903032), the Hunan Provincial Natural Science Foundation of China (No. 2021JJ41013), the China Postdoctoral Science Foundation (No. 2020M672520) and the Research Program of Hunan Health Commission, China (No. 202103030659).

## Conflict of Interest

The authors declare that the research was conducted in the absence of any commercial or financial relationships that could be construed as a potential conflict of interest.

## Publisher's Note

All claims expressed in this article are solely those of the authors and do not necessarily represent those of their affiliated organizations, or those of the publisher, the editors and the reviewers. Any product that may be evaluated in this article, or claim that may be made by its manufacturer, is not guaranteed or endorsed by the publisher.

## References

[1] Vincent A Herman J Schulick R Hruban RH Goggins M. Pancreatic cancer. Lancet. (2011) 378:607–20. 10.1016/S0140-6736(10)62307-0 21620466 PMC3062508

[2] Wu M Li X Zhang T Liu Z Zhao Y. Identification of a nine-gene signature and establishment of a prognostic nomogram predicting overall survival of pancreatic cancer. Front Oncol. (2019) 9:996. 10.3389/fonc.2019.00996 31612115 PMC6776930

[3] Chu LC Goggins MG Fishman EK. Diagnosis and detection of pancreatic cancer. Cancer J. (2017) 23:333–42. 10.1097/PPO.0000000000000290 29189329

[4] Wolpin BM. Pancreatic cancer. Hematol Oncol Clin North Am. (2015) 29:xiii–xiv. 10.1016/j.hoc.2015.06.002 26226911

[5] Saluja A Maitra A. Pancreatitis and pancreatic cancer. Gastroenterology. (2019) 156:1937–40. 10.1053/j.gastro.2019.03.050 30940522

[6] Wang Y Nie H He X Liao Z Zhou Y Zhou J. The emerging role of super enhancer-derived noncoding RNAs in human cancer. Theranostics. (2020) 10:11049–62. 10.7150/thno.49168 33042269 PMC7532672

[7] Zhou J Wu G Tong Z Sun J Su J Cao Z. Prognostic relevance of SMC family gene expression in human sarcoma. Aging. (2020) 13:1473–87. 10.18632/aging.202455 33460400 PMC7835044

[8] Nie H Wang Y Yang X Liao Z He X Zhou J. Clinical significance and integrative analysis of the SMC family in hepatocellular carcinoma. Front Med. (2021) 8:727965. 10.3389/fmed.2021.727965 34527684 PMC8437102

[9] Schalbetter SA Goloborodko A Fudenberg G Belton JM Miles C Yu M. SMC complexes differentially compact mitotic chromosomes according to genomic context. Nat Cell Biol. (2017) 19:1071–80. 10.1038/ncb3594 28825700 PMC5640152

[10] Uhlmann F. SMC complexes: from DNA to chromosomes. Nat Rev Mol Cell Biol. (2016) 17:399–412. 10.1038/nrm.2016.30 27075410

[11] Muir KW Li Y Weis F Panne D. The structure of the cohesin ATPase elucidates the mechanism of SMC-kleisin ring opening. Nat Struct Mol Biol. (2020) 27:233–9. 10.1038/s41594-020-0379-7 32066964 PMC7100847

[12] Wang Q Wang C Li N Liu X Ren W Wang Q. Condensin Smc4 promotes inflammatory innate immune response by epigenetically enhancing NEMO transcription. J Autoimmun. (2018) 92:67–76. 10.1016/j.jaut.2018.05.004 29803706

[13] Pandey R Abel S Boucher M Wall RJ Zeeshan M Rea E. Plasmodium condensin core subunits SMC2/SMC4 mediate atypical mitosis and are essential for parasite proliferation and transmission. Cell Rep. (2020) 30:1883–97 e6. 10.1016/j.celrep.2020.01.033 32049018 PMC7016506

[14] Roy MA Dhanaraman T D'Amours D. The Smc5-Smc6 heterodimer associates with DNA through several independent binding domains. Sci Rep. (2015) 5:9797. 10.1038/srep09797 25984708 PMC4434891

[15] Torres-Rosell J Machin F Farmer S Jarmuz A Eydmann T Dalgaard JZ. SMC5 and SMC6 genes are required for the segregation of repetitive chromosome regions. Nat Cell Biol. (2005) 7:412–9. 10.1038/ncb1239 15793567

[16] Li K Ying M Feng D Chen Y Wang J Wang Y. SMC1 promotes epithelial-mesenchymal transition in triple-negative breast cancer through upregulating Brachyury. Oncol Rep. (2016) 35:2405–12. 10.3892/or.2016.4564 26781859

[17] Feng Y Liu H Duan B Liu Z Abbruzzese J Walsh KM. Potential functional variants in SMC2 and TP53 in the AURORA pathway genes and risk of pancreatic cancer. Carcinogenesis. (2019) 40:521–8. 10.1093/carcin/bgz029 30794721 PMC6556704

[18] Montero S Seras-Franzoso J Andrade F Martinez-Trucharte F Vilar-Hernandez M Quesada M. Intracellular delivery of Anti-SMC2 antibodies against cancer stem cells. Pharmaceutics. (2020) 12:185. 10.3390/pharmaceutics12020185 32098204 PMC7076674

[19] Chin CV Antony J Ketharnathan S Labudina A Gimenez G Parsons KM. Cohesin mutations are synthetic lethal with stimulation of WNT signaling. Elife. (2020) 9:e61405. 10.7554/eLife.61405 33284104 PMC7746233

[20] Zhang C Kuang M Li M Feng L Zhang K Cheng S. SMC4, which is essentially involved in lung development, is associated with lung adenocarcinoma progression. Sci Rep. (2016) 6:34508. 10.1038/srep34508 27687868 PMC5043270

[21] Hamada K Ueda M Satoh M Inagaki N Shimada H Yamada-Okabe H. Increased expression of the genes for mitotic spindle assembly and chromosome segregation in both lung and pancreatic carcinomas. Cancer Genomics Proteomics. (2004) 1:231–40. 31394658

[22] Tang Z Li C Kang B Gao G Li C Zhang Z. A web server for cancer and normal gene expression profiling and interactive analyses. Nucleic Acids Res. (2017) 45:W98–102. 10.1093/nar/gkx247 28407145 PMC5570223

[23] Navani S. Manual evaluation of tissue microarrays in a high-throughput research project: the contribution of Indian surgical pathology to the human protein atlas (HPA) project. Proteomics. (2016) 16:1266–70. 10.1002/pmic.201500409 26748468

[24] Hou GX Liu P Yang J Wen S. Mining expression and prognosis of topoisomerase isoforms in non-small-cell lung cancer by using oncomine and kaplan-meier plotter. PLoS ONE. (2017) 12:e0174515. 10.1371/journal.pone.0174515 28355294 PMC5371362

[25] Cerami E Gao J Dogrusoz U Gross BE Sumer SO Aksoy BA. The cBio cancer genomics portal: an open platform for exploring multidimensional cancer genomics data. Cancer Discov. (2012) 2:401–4. 10.1158/2159-8290.CD-12-0095 22588877 PMC3956037

[26] Wang Y Nie H Liao Z He X Xu Z Zhou J. Expression and clinical significance of lactate dehydrogenase A in colon adenocarcinoma. Front Oncol. (2021) 11:700795. 10.3389/fonc.2021.700795 34307169 PMC8300199

[27] Li T Fan J Wang B Traugh N Chen Q Liu JS. TIMER: a web server for comprehensive analysis of tumor-infiltrating immune cells. Cancer Res. (2017) 77:e108–e10. 10.1158/0008-5472.CAN-17-0307 29092952 PMC6042652

[28] Griffiths S. Radiotherapy quality control–portal and verification films. Radiogr Today. (1990) 56:17. 2363821

[29] Li J He J Wang Y Shu Y Zhou J. SMC1 promotes proliferation and inhibits apoptosis through the NFkappaB signaling pathway in colorectal cancer. Oncol Rep. (2019) 42:1329–42. 10.3892/or.2019.7273 31524239 PMC6718103

[30] Zhang Y Yi F Wang L Wang Z Zhang N Wang Z. Phosphorylation of SMC1A promotes hepatocellular carcinoma cell proliferation and migration. Int J Biol Sci. (2018) 14:1081–9. 10.7150/ijbs.24692 29988990 PMC6036730

[31] Homme C Krug U Tidow N Schulte B Kuhler G Serve H. Low SMC1A protein expression predicts poor survival in acute myeloid leukemia. Oncol Rep. (2010) 24:47–56. 10.3892/or_00000827 20514443

[32] Han YH Wan Y Xiong H Sun GL. Structural maintenance of chromosomes 2 is identified as an oncogene in bladder cancer *in vitro* and *in vivo*. Neoplasma. (2020) 67:364–70. 10.4149/neo_2020_190510N419 31986889

[33] Wang D Wang L Zhang Y Zhao Y Chen G. Hydrogen gas inhibits lung cancer progression through targeting SMC3. Biomed Pharmacother. (2018) 104:788–97. 10.1016/j.biopha.2018.05.055 29852353

[34] Kraft B Lombard J Kirsch M Wuchter P Bugert P Hielscher T. SMC3 protein levels impact on karyotype and outcome in acute myeloid leukemia. Leukemia. (2019) 33:795–9. 10.1038/s41375-018-0287-6 30323357

[35] Jiang L Zhou J Zhong D Zhou Y Zhang W Wu W. Overexpression of SMC4 activates TGFbeta/Smad signaling and promotes aggressive phenotype in glioma cells. Oncogenesis. (2017) 6:e301. 10.1038/oncsis.2017.8 28287612 PMC5533949

[36] Huang T Xiang J Wang Y Tuo Y. Changes of EGFR and SMC4 expressions in triple-negative breast cancer and their early diagnostic value. Gland Surg. (2021) 10:1118–24. 10.21037/gs-21-119 33842255 PMC8033084

[37] Potts PR Yu H. The SMC5/6 complex maintains telomere length in ALT cancer cells through SUMOylation of telomere-binding proteins. Nat Struct Mol Biol. (2007) 14:581–90. 10.1038/nsmb1259 17589526

[38] Riviere L Quioc-Salomon B Fallot G Halgand B Feray C Buendia MA. Hepatitis B virus replicating in hepatocellular carcinoma encodes HBx variants with preserved ability to antagonize restriction by Smc5/6. Antiviral Res. (2019) 172:104618. 10.1016/j.antiviral.2019.104618 31600532

[39] Decorsiere A Mueller H van Breugel PC Abdul F Gerossier L Beran RK. Hepatitis B virus X protein identifies the Smc5/6 complex as a host restriction factor. Nature. (2016) 531:386–9. 10.1038/nature17170 26983541

[40] Morrison AH Byrne KT Vonderheide RH. Immunotherapy and prevention of pancreatic cancer. Trends Cancer. (2018) 4:418–28. 10.1016/j.trecan.2018.04.001 29860986 PMC6028935

[41] Nie H Wang Y Liao Z Zhou J Ou C. The function and mechanism of circular RNAs in gastrointestinal tumours. Cell Prolif. (2020) 53:e12815. 10.1111/cpr.12815 32515024 PMC7377939

[42] Winograd R Byrne KT Evans RA Odorizzi PM Meyer AR Bajor DL. Induction of T-cell immunity overcomes complete resistance to PD-1 and CTLA-4 blockade and improves survival in pancreatic carcinoma. Cancer Immunol Res. (2015) 3:399–411. 10.1158/2326-6066.CIR-14-0215 25678581 PMC4390506

[43] Spear S Candido JB McDermott JR Ghirelli C Maniati E Beers SA. Discrepancies in the tumor microenvironment of spontaneous and orthotopic murine models of pancreatic cancer uncover a new immunostimulatory phenotype for B cells. Front Immunol. (2019) 10:542. 10.3389/fimmu.2019.00542 30972056 PMC6445859

[44] Peng L Wang D Han Y Huang T He X Wang J. Emerging role of cancer associated fibroblasts-derived exosomes in tumorigenesis. Front Immunol. (2022) 12:795372. 10.3389/fimmu.2021.795372 35058933 PMC8764452

[45] Keenan BP Saenger Y Kafrouni MI Leubner A Lauer P Maitra A. A listeria vaccine and depletion of T-regulatory cells activate immunity against early stage pancreatic intraepithelial neoplasms and prolong survival of mice. Gastroenterology. (2014) 146:1784–94 e6. 10.1053/j.gastro.2014.02.055 24607504 PMC4035450

[46] Okubo S Suzuki T Hioki M Shimizu Y Toyama H Morinaga S. The immunological impact of preoperative chemoradiotherapy on the tumor microenvironment of pancreatic cancer. Cancer Sci. (2021). 10.1111/cas.14914 33931909 PMC8253289

